# Pragmatic Clinical Trial of Fostamatinib as Second‐Line Therapy in Adult Patients With Immune Thrombocytopenia and Insufficient Response to Prior Therapy: The Fostamatinib FORTE Trial

**DOI:** 10.1002/jha2.70355

**Published:** 2026-07-11

**Authors:** Amber Afzal, Robert Kribs, Sumit Sawhney, Mohit Narang, Frances Blevins, Robert Numerof, Francois Di Trapani, Sherry Ladha, Ruchika Goel

**Affiliations:** ^1^ Department of Medicine Washington University School of Medicine St. Louis Missouri USA; ^2^ Hematologic Malignancies and Cellular Therapeutics University of Kansas Health System Westwood Kansas USA; ^3^ Department of Internal Medicine HCA East Florida Fort Lauderdale Florida USA; ^4^ Maryland Oncology Hematology US Oncology Research Columbia Maryland USA; ^5^ Center for Early Detection and Interception of Blood Cancers Dana‐Farber Cancer Institute Boston Massachusetts USA; ^6^ Rigel Pharmaceuticals, Inc. South San Francisco California USA; ^7^ Departments of Internal Medicine and Pediatrics Simmons Cancer Institute SIU School of Medicine Springfield Illinois USA

**Keywords:** bleeding, clinical trial, fostamatinib, immune thrombocytopenia, responder, second‐line therapy

1

Primary immune thrombocytopenia (ITP) is an autoimmune disease with increased risk of bleeding due to antibody‐mediated platelet destruction and impaired platelet production [1]. Treatment goals include achieving adequate platelet counts while minimizing treatment toxicity to reduce bleeding risk and improve health‐related quality of life (HRQoL) [2].

Short‐course corticosteroid therapy is the standard‐of‐care first‐line treatment for ITP [3, 4]. If corticosteroids are intolerable or ineffective, intravenous immunoglobulin or anti‐D treatment is recommended [4]. Pharmacological treatments approved for second‐line therapy by the US Food and Drug Administration (FDA) include fostamatinib, rilzabrutinib, and thrombopoietin receptor agonists (TPO‐RAs). Treatment guidelines also recommend rituximab [3, 4].

Fostamatinib, a spleen tyrosine kinase (SYK) inhibitor, acts as an immunomodulatory agent by targeting antibody‐mediated destruction of platelets [5]. Among patients with persistent/chronic ITP enrolled in two randomized placebo‐controlled Phase 3 trials (NCT02076399 and NCT02076412), those treated with fostamatinib had a significantly greater response rate (43/101 [43%] had ≥ 1 platelet count ≥ 50 × 10^9^/L) and stable response rate (18/101 [18%] had platelet counts ≥ 50 × 10^9^/L on ≥ 4/6 visits) versus placebo (7/49 [14%] and 1/49 [2%], respectively; both *p *< 0.01) [6]. Fostamatinib treatment was associated with fewer bleeding events and increased incidence of diarrhea, hypertension, and nausea versus placebo [5]. A post hoc analysis of patients treated with fostamatinib in second‐line (*n* = 32) and third‐line or later (*n* = 113) settings reported response rates of 78% and 48%, with responses maintained for a median of 83% and 86% of treatment days, respectively [7].

This pragmatic, single‐arm study (FORTE) prospectively explored the effectiveness and safety of fostamatinib when used as second‐line therapy for ITP (Supporting Information). Patients initiating fostamatinib per treating clinician's decision signed informed consent and were followed for 12 months. End points included platelet count measures, concomitant ITP‐related medication use, adverse events (AEs), and HRQoL via the ITP Patient Assessment Questionnaire (ITP‐PAQ). The full analysis population included patients who received ≥ 1 dose of fostamatinib and had ≥ 1 follow‐up assessment.

The sponsor terminated the study on November 30, 2022, because of slow enrollment related to the COVID‐19 pandemic. Sixteen patients were enrolled; 15 received fostamatinib, comprising the full analysis population (Table S1; Figure S1). The median (interquartile range [IQR]) age was 48 (27, 63) years. Eight (53.3%) patients had thromboembolic risk factors at baseline; nine (60.0%) patients were taking corticosteroids at the time of the first fostamatinib dose. The most common schedule was fostamatinib 100 mg twice daily (Table S2).

Median (IQR) platelet counts increased from 43 × 10^9^/L (19.0–105.0) at baseline (*n* = 13) to 167 × 10^9^/L (96.0–180.0) at end of study (*n* = 6), with similar increases irrespective of steroid use at baseline (Figure 1). The median (range) cumulative duration of sustained platelet counts between 30 and 450 × 10^9^/L was 31.7 (1–53) weeks. At least one platelet count > 50 × 10^9^/L was observed in 14/15 (93.3%) patients.

**FIGURE 1:**
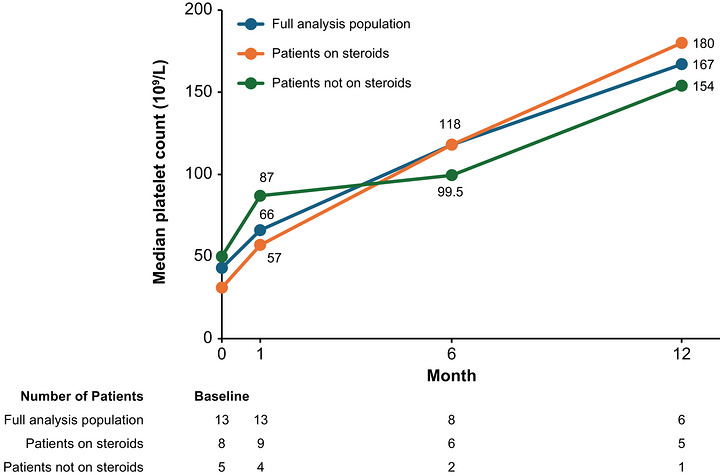
Median platelet counts over time. The full analysis population included 15 patients who received at least 1 dose of fostamatinib after giving written informed consent and had at least 1 follow‐up assessment collected. The “Patients on steroids” and “Patients not on steroids” categories are based on whether or not a patient was receiving steroids prior to the first dose of fostamatinib.

The median (IQR) dose of maintenance corticosteroids (prednisone) steadily decreased from 40.0 (30.0–60.0) mg/day (*n* = 7) at baseline to 5.0 (5.0–5.0) mg/day (*n* = 1) at Month 6. No patients were taking maintenance corticosteroids at Months 9 or 12 (Figure 2). Minimal rescue medication was needed: 1/15 patient at baseline, 2/15 at Month 1, 1/15 at Month 3, and 1/6 at Month 12.

**FIGURE 2:**
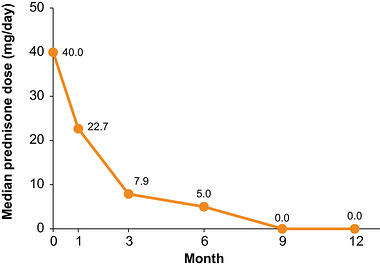
Tapering of maintenance prednisone after fostamatinib initiation.

Among five patients with ITP‐PAQ assessments following ≥ 36 weeks of treatment, significant least‐squares mean (LSM) improvements from baseline were observed for 7/10 ITP‐PAQ domain scores (all *p *< 0.05), including overall QoL (Table S3). A significant LSM (SE) improvement from baseline in ITP‐PAQ item 10 (i.e., physical fatigue) score was also observed at 36–54 weeks (38.9 [10.1]; *p *< 0.02).

Four patients discontinued the study due to AEs (Figure S1; Table S4). Five patients reported eight bleeding‐related AEs, all of which were mild or moderate. Two patients experienced serious AEs: one hospitalization for Grade 2 dyspnea and chills unrelated to fostamatinib, and one hospitalization for Grade 3 diarrhea possibly related to fostamatinib.

In summary, median platelet counts of patients with ITP increased over 12 months of second‐line fostamatinib treatment, with 14/15 patients experiencing platelet counts > 50 × 10^9^/L. In one patient, fostamatinib dose could be reduced due to stabilization of platelet counts. No FORTE patients remained on corticosteroids by Month 9 of treatment. Patients with ITP experience significantly reduced HRQoL, especially fatigue, which may not improve with increases in platelet counts [2]. Here, clinically meaningful improvements from baseline were observed in 7/8 ITP‐PAQ domains with established minimal important difference (MID) thresholds, including fatigue/sleep, following ≥ 36 weeks of fostamatinib treatment. Drug‐related serious AEs were rarer in FORTE than in the second‐line treatment group detailed in Boccia et al. [7] (13% vs. 34%). Rates of bleeding events in this study are comparable to those reported for other ITP‐directed therapies, such as romiplostim (32%) and eltrombopag (27%) [8].

In contrast to fostamatinib, 35% of the patients on avatrombopag [9] and 82% on eltrombopag [10] were able to discontinue steroids in real‐world analyses. Similarly, among patients treated with rilzabrutinib, clinically meaningful improvements in HRQoL were observed in only half of the ITP‐PAQ domains with established MID thresholds, including fatigue/sleep [11].

Furthermore, fostamatinib has a favorable thromboembolic risk profile. TPO‐RAs increase thrombotic risk [12]. Long‐term studies report annualized incidence rates of 4%–7% with TPO‐RAs, which is 2–3 times higher than among ITP patients not receiving TPO‐RAs [13, 14]. No increase in thromboembolic events has previously been observed with fostamatinib. Although eight (53.3%) patients entered FORTE with ≥ 1 thromboembolic risk factor, no serious AEs related to thrombotic events were reported. This result may relate to fostamatinib's mechanism of action [12]. SYK controls signaling of receptors involved in thrombus formation, including FcyRIIA gamma‐globulin, glycoprotein VI, and C‐type lectin‐like II (CLEC‐2) receptors. By inhibiting SYK, fostamatinib selectively targets these pathways and spares pathways that maintain hemostasis, reducing risk of bleeding [12].

This study was limited by the small number of enrolled patients and lack of a comparator group. The ITP‐PAQ outcomes are subject to potential selection and responder bias, as later‐week estimates included only patients still on treatment. However, the clinically meaningful increases in platelet counts and reductions in maintenance corticosteroid use in > 90% of patients support the real‐world effectiveness of second‐line fostamatinib for ITP.

## Funding

This study was funded by Rigel Pharmaceuticals, Inc.

## Ethics Statement

The study was conducted in compliance with the ethical principles in the Declaration of Helsinki, all applicable requirements from local regulatory authorities, the US Food and Drug Administration Code of Federal Regulations (CFR), 21 CFR Part 50 and Part 312, and in accordance with International Council for Harmonization of Technical Requirements for Pharmaceuticals for Human Use Good Clinical Practice. Approval was obtained from the Institutional Review Board for the study protocol, as well as for any amendments, informed consent forms, and any revised consent forms, patient recruitment documents, and any other study documentation provided to patients.

## Consent

Participants provided written informed consent following eligibility screening and prior to treatment initiation.

## Conflicts of Interest

Amber Afzal received an ISTH Large Grant in 2025. Mohit Narang has a consulting/advisory role for Celgene and Bristol Myers Squibb and has served on a speakers’ bureau for Bristol Myers Squibb. Robert Numerof, Francois Di Trapani, and Sherry Ladha have employment and stock ownership in Rigel Pharmaceuticals, Inc. Ruchika Goel has a consulting/advisory role with Rigel Pharmaceuticals, Inc. The other authors declare no conflicts of interest.

## Supporting information




**Supporting Information**: jha270355‐sup‐0001‐SuppMat.docx

## Data Availability

For deidentified data, requests may be sent to datasharing@rigel.com at least 24 months after clinical trial completion, provided a scientifically valid research proposal is made by qualified academic researchers for data associated with interventions that have received regulatory approval in the US and Europe.
