# Supplementary material for: Pragmatic Clinical Trial of Fostamatinib as Second‐Line Therapy in Adult Patients With Immune Thrombocytopenia and Insufficient Response to Prior Therapy: The Fostamatinib FORTE Trial
Source: EJHaem. 2026 Jul 11;7(4):e70355. doi: 10.1002/jha2.70355 (PMC13354938; doi:10.1002/jha2.70355)
Supplement: Supplementary file 1 — Supporting Information: jha270355‐sup‐0001‐SuppMat.docx [file JHA2-7-e70355-s001.docx]

**Supplementary Files**

**Supplementary Methods**

FORTE was a prospective, pragmatic, single-arm, study of fostamatinib as second-line therapy conducted at 14 sites in the US. Cohort 1 was to include approximately 30 patients who initiated fostamatinib as second-line therapy. A second cohort, meant to include patients already on fostamatinib for ≥12 weeks as second-line therapy at time of enrollment, did not enroll any patients. Inclusion criteria specified adult patients (≥18 years old) who had an insufficient response to steroids with or without intravenous immunoglobulins. Patients who had prior ITP therapy other than steroids and/or intravenous immunoglobulins, who planned to participate in an interventional study, who lacked historical platelet counts, or who were pregnant/nursing were excluded. Patients were enrolled in the study after providing written informed consent and were followed for a maximum of 12 months.

The choice to initiate treatment with fostamatinib as second-line therapy was made by the treating clinician independent of, and prior to, a decision to enroll the patient into the study. The study followed a pragmatic design with standard-of-care office visits and laboratory draws to collect and analyze data on effectiveness, safety, and dose adjustments for fostamatinib.

Study end points included fostamatinib dosing and changes in dosing, platelet counts over time, the duration of elevated platelet levels (durability of response), the number of patients with a postbaseline platelet count >30×10^9^/L, and the number of patients with a postbaseline platelet count >50×10^9^/L. The use of concomitant ITP treatment, including changes in doses over time that were due to rescue medication use and/or initiation of another ITP therapy were also assessed. The duration of elevated platelet counts was measured from the time of first platelet count >30×10^9^/L until first subsequent platelet count ≤30×10^9^/L or >450×10^9^/L. The median cumulative duration of elevated platelet counts was defined as the sum of the durations of time that a platelet count was between 30×10^9^/L and 450×10^9^/L since the date of the first use of fostamatinib. Quality of life was assessed by the ITP Patient Assessment Questionnaire (ITP-PAQ) at enrollment, first standard of care follow-up visit, 6 months after enrollment, and end of study.^1^ Incidence of ITP-related AEs (including all bleeding events or worsening of ITP) and serious AEs were also reported.

Continuous data were summarized using descriptive statistics. Categorical data were described using frequencies and percentages. Least-squares means (LSMs) of ITP-PAQ domain scores were calculated using a linear mixed-effects model adjusting for baseline score and grouped study week, with subject as a random effect. Significance was determined using Tukey-adjusted pairwise comparisons of LSMs. Quality-of-life outcomes were analyzed using R v4.5.1. All other statistical analyses were performed using SAS® v9.4 or above. The Full Analysis Population consisted of all enrolled patients who received at least 1 dose of fostamatinib and had at least 1 follow-up assessment collected; patient demographics, dosing, and effectiveness were reported using this population. Additional analyses included subgroups of patients either taking or not taking steroids at the time of the first dose of fostamatinib. The Safety Population consisted of all enrolled patients who received at least 1 dose of fostamatinib after giving written informed consent and was identical to the Full Analysis Population. AE data are reported for this population.

The study was conducted in compliance with the ethical principles in the Declaration of Helsinki, all applicable requirements from local regulatory authorities, and the US Food and Drug Administration Code of Federal Regulations (CFR), 21 CFR Part 50 and Part 312 and in accordance with the International Council for Harmonization of Technical Requirements for Pharmaceuticals for Human Use Good Clinical Practice. Approval was obtained from the Institutional Review Board for the study protocol, any amendments, informed consent forms and any revised consent forms, patient recruitment documents, and any other study documentation provided to patients.

**Supplementary Table 1.** Baseline Demographics and Disease Characteristics

| Category | Full Analysis Population (N=15) |
| --- | --- |
| Age at time of informed consent, years, median (IQR) | 48 (27.0-63.0) |
| Sex, n (%) |  |
| Male | 6 (40.0) |
| Female | 9 (60.0) |
| Race, n (%) |  |
| White | 10 (66.7) |
| Other/unknown | 5 (33.3) |
| Ethnicity, n (%) |  |
| Hispanic or Latino | 5 (33.3) |
| Not Hispanic or Latino | 10 (66.7) |
| Age at ITP diagnosis, years, median (IQR) | 40 (27.0-62.0) |
| Thromboembolic risk factor, n (%)^a^ |  |
| Yes | 8 (53.3) |
| Prior ITP-related medication use, n (%) |  |
| Yes | 13 (86.7) |
| Steroids NOS | 1 (6.7) |
| Dexamethasone | 5 (33.3) |
| Prednisone | 8 (53.3) |
| Immunoglobulin G, human | 1 (6.7) |
| Immunoglobulin, human normal | 2 (13.3) |
| Immunoglobulins NOS | 3 (20.0) |

^a^Thromboembolic risk factors included obesity, cancer, hyperlipidemia, diabetes, hypertension, coronary disease, chronic kidney disease, and presence of antiphospholipid antibodies.

Abbreviations: IQR, interquartile range; ITP, immune thrombocytopenia; NOS, not otherwise specified.

**Supplementary Table 2.** Fostamatinib Dosing and Changes in Dosing

| **Category** | **Baseline**  **(n=5)** | **Month 1**  **(n=15)** | **Month 3**  **(n=13)** | **Month 6**  **(n=8)** | **Month 9**  **(n=7)** | **Month 12**  **(n=6)** |
| --- | --- | --- | --- | --- | --- | --- |
| **Total daily dose (mg), median (IQR)^a^** | 200.0  (200.0, 200.0) | 200.0  (200.0, 200.0) | 200.0  (200.0, 296.7) | 200.0  (200.0, 267.4) | 200.0  (200.0, 300.0) | 250.0  (200.0, 300.0) |
| **Dosage and frequency, n (%)** |  |  |  |  |  |  |
| 100 mg every other day | 0 | 0 | 1 (6.7) | 1 (6.7) | 1 (6.7) | 0 |
| 100 mg QD | 0 | 1 (6.7) | 1 (6.7) | 0 | 1 (6.7) | 1 (6.7) |
| 150 mg QD | 0 | 0 | 1 (6.7) | 0 | 0 | 0 |
| 100 mg BID | 5 (33.3) | 15 (100.0) | 9 (60.0) | 5 (33.3) | 3 (20.0) | 3 (20.0) |
| 150 mg BID | 0 | 3 (20.0) | 4 (26.7) | 3 (20.0) | 3 (20.0) | 3 (20.0) |
| **Reason for dose adjustment^b^** |  |  |  |  |  |  |
| Adverse event | – | 1 (25.0) | 0 | 0 | 0 | 0 |
| Investigator decision | – | 0 | 0 | 1 (100.0) | 0 | 0 |
| Lack of effectiveness | – | 3 (75.0) | 1 (50.0) | 0 | 1 (100.0) | 1 (100.0) |
| Other | – | 0 | 2 (100.0) | 0 | 0 | 0 |
| Platelets stable, tapering drug | – | 0 | 1 (50.0) | 0 | 0 | 0 |
| Stable platelets | – | 0 | 1 (50.0) | 0 | 0 | 0 |

^a^If a patient has multiple records of fostamatinib use at the same time point, only the average dosage of all the records for that patient is reported.

^b^A patient can have multiple instances of dose adjustments at the same time point.

Abbreviations: BID, twice daily; IQR, interquartile range; QD, once daily.

**Supplementary Table 3.** ITP-PAQ Scores by Grouped Study Week

| **Domain** | **MID^2^** | **1-8 weeks**  **(n=9)** | **19-28 weeks**  **(n=4)** | **36-54 weeks**  **(n=5)** |
| --- | --- | --- | --- | --- |
| **Mean (SE) change from baseline** |  |  |  |  |
| Overall QoL | 8-12 | **14.1 (8.2)** | **37.5 (21.6)** | **52.0 (15.8)** |
| Symptoms | 8-12 | -1.4 (5.6) | **13.8 (15.3)** | **22.5 (16.0)** |
| Bother-Physical Health | 8-12 | **14.5 (8.1)** | **43.1 (8.4)** | **52.8 (17.9)** |
| Fatigue/Sleep | 10-15 | 3.0 (6.3) | 6.8 (11.1) | **27.5 (15.3)** |
| Item 10 (physical fatigue)^b^ | **–** | 6.3 (9.2) | 0 (28.9) | **40.0 (18.7)^a^** |
| Activity | 10-15 | **16.7 (13.2)** | **37.5 (22.2)** | **57.5 (22.2)** |
| Fear | ND | -0.6 (6.5) | 30.0 (12.1) | 43.0 (14.5) |
| Psychological Health | 8-12 | 5.0 (9.8) | **22.5 (20.5)** | **28.0 (13.4)** |
| Work | ND | 7.2 (5.2) | 15.0 (10.6) | 25.0 (8.8) |
| Social Activity | 8-12 | 9.7 (6.4) | **29.7 (12.1)** | **45.0 (20.5)** |
| Women’s Reproductive Health^c^ | 8-12 | -5.8 (8.1) | **18.1 (14.1)** | 0 (0) |
| **LSM (SE) change from baseline** |  |  |  |  |
| Overall QoL | **–** | 14.0 (6.9) | 36.9 (10.8)* | 48.1 (10.0)** |
| Symptoms | **–** | -1.4 (5.7) | 14.9 (8.9) | 21.9 (8.2) |
| Bother-Physical Health | **–** | 14.9 (6.1) | 41.4 (9.6)** | 49.1 (8.8)*** |
| Fatigue/Sleep | **–** | 2.7 (5.0) | 6.2 (7.9) | 27.3 (7.3)* |
| Item 10 (physical fatigue)^b^ | **–** | 6.0 (7.3) | 1.2 (12.8) | 38.9 (10.1)* |
| Activity | **–** | 16.6 (9.2) | 36.5 (14.7) | 53.5 (13.5)* |
| Fear | **–** | -0.6 (5.6) | 29.8 (8.9)* | 38.5 (8.2)** |
| Psychological Health | **–** | 4.3 (6.7) | 23.9 (10.5) | 23.8 (9.8) |
| Work | **–** | 7.7 (4.0) | 13.6 (6.1) | 23.4 (5.7)* |
| Social Activity | **–** | 9.9 (6.3) | 26.7 (10.1) | 40.2 (9.2)** |
| Women’s Reproductive Health^c^ | **–** | -6.1 (6.7) | 18.8 (9.1) | -0.6 (11.5) |

Bold values exceed the MID. **P*<0.05, ***P*<0.01, ****P*<0.001.

^a^Compared to the MID for the Fatigue/Sleep domain.

^b^Item 10 (physical fatigue) was reported in 8 patients (1-8 weeks), 3 patients (19-28 weeks), and 5 patients (36-54 weeks).

^c^Women’s Reproductive Health was reported in 5 patients (1-8 weeks), 3 patients (19-28 weeks), and 2 patients (36-54 weeks).

Abbreviations: ITP-PAQ, immune thrombocytopenia Patient Assessment Questionnaire; LSM, least squares mean; MID, minimal important difference; ND, not determined; QoL, quality of life; SE, standard error.

**Supplementary Table 4.** Summary of AEs

| **Category, n (%)** | **Safety Population (N=15)** |
| --- | --- |
| **Bleeding-related events** |  |
| Gingival bleeding | 2 (13.3) |
| Mucosal hemorrhage | 2 (13.3) |
| Epistaxis | 1 (6.7) |
| **Serious AEs (n=2)** |  |
| Dyspnea | 1 (6.7)^a^ |
| Chills | 1 (6.7)^a^ |
| Diarrhea | 1 (6.7)^b^ |
| **Treatment-related AEs leading to study discontinuation (n=4)** |  |
| Diarrhea | 1 (6.7)^b^ |
| Noncardiac chest pain | 1 (6.7) |
| Fatigue | 1 (6.7) |
| Pancreatitis | 1 (6.7) |

^a^Dyspnea and chills were reported in the same patient.

^b^Diarrhea reported in 1 unique patient was a serious AE that also led to study discontinuation.

Abbreviations: AE, adverse event.

**Supplementary Figure 1.** Patient Disposition


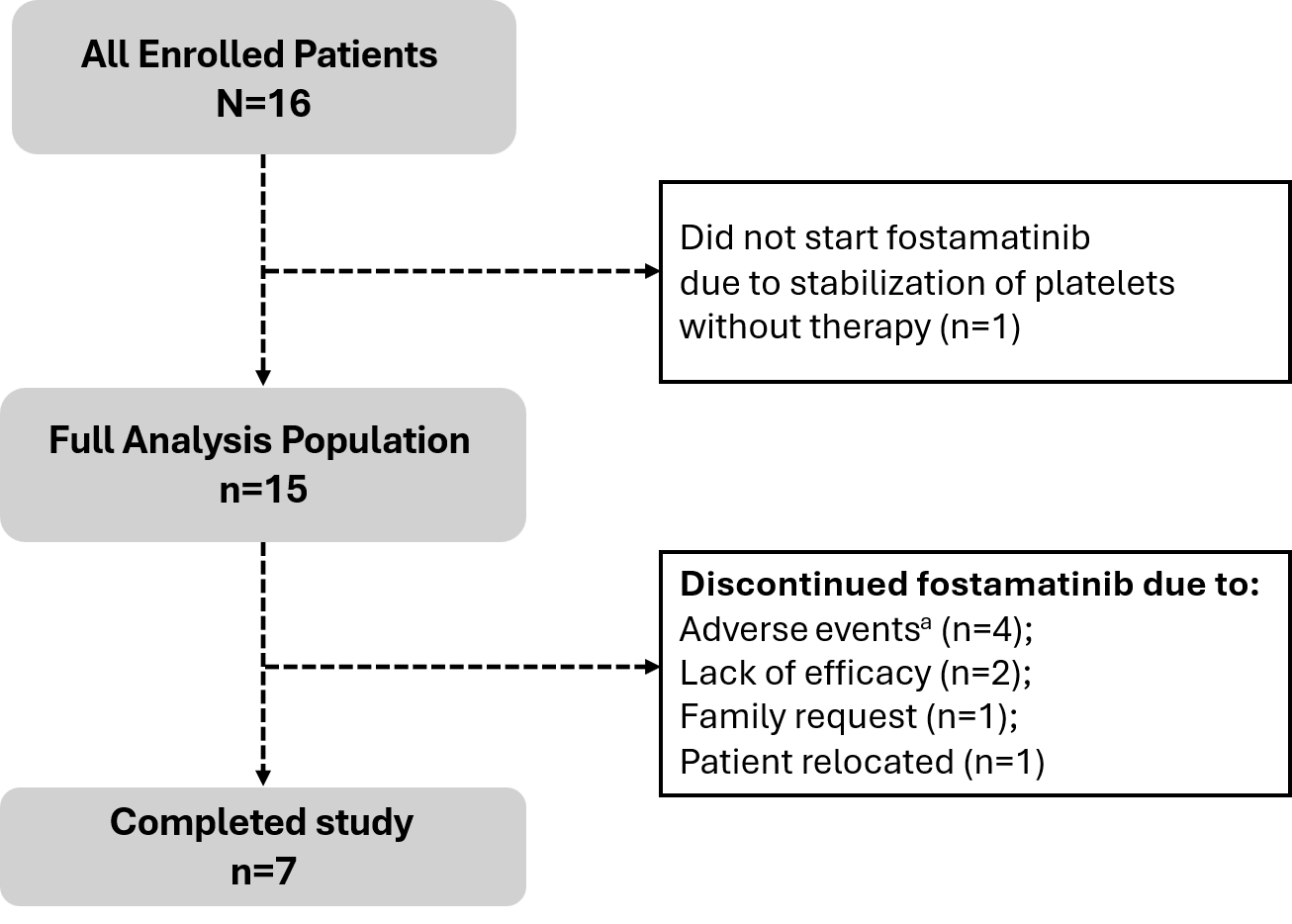


^a^Adverse events resulting in discontinuation included 1 case each of grade 2 noncardiac chest pain, grade 2 fatigue, grade 1 pancreatitis, and grade 3 serious AE of diarrhea. All were assessed as possibly related to fostamatinib, and all resolved following study discontinuation.

**Supplementary References**

1. Mathias SD, Bussel JB, George JN, et al. A disease-specific measure of health-related quality of life for use in adults with immune thrombocytopenic purpura: its development and validation. *Health Qual Life Outcomes.* 2007;5:11. <https://doi.org/10.1186/1477-7525-5-11>.

2. Mathias SD, Gao SK, Rutstein M, et al. Evaluating clinically meaningful change on the ITP-PAQ: preliminary estimates of minimal important differences. *Curr Med Res Opin.* 2009;25(2):375-383. https://doi.org/10.1185/03007990802634119.
